# Effect of a free sugar-restricted diet on anthropometric parameters, lipid profile, and glycemic control in women with polycystic ovary syndrome: a randomized controlled trial

**DOI:** 10.1186/s12902-026-02451-3

**Published:** 2026-08-04

**Authors:** Hajar Gholamshahi, Alireza Houshdar Tehrani, Danial Fotros, Asal Ataie-Jafari

**Affiliations:** 1 Department of Nutrition, SR.C., Islamic Azad University, Tehran, Iran; 2 School of Medicine, Shahid Beheshti University of Medical Sciences, Tehran, Iran; 3 Department of Nutritional Sciences, College of Human Sciences, Texas Tech University, Lubbock, TX 79409 USA

**Keywords:** Polycystic ovary syndrome, Insulin resistance, Lipid profile, Free sugar-restricted diet

## Abstract

**Background:**

High-sugar diets rich in fructose and other simple sugars are associated with increased lipogenesis and insulin resistance, potentially increasing the risk of metabolic syndrome, a condition closely linked with polycystic ovary syndrome (PCOS). This study aimed to evaluate the effects of a free sugar-restricted diet on anthropometric parameters, lipid profile, and glycemic control in women with PCOS.

**Methods:**

In this randomized controlled clinical trial, 74 women aged 18–45 years with diagnosed PCOS were randomly assigned to either a free sugar-restricted diet group (n = 37) or a standard diet group (n = 37) for 12 weeks. Anthropometric indices, fasting blood glucose (FBG), serum insulin, insulin (HOMA-IR), insulin sensitivity (QUICKI), and lipid profile parameters including triglycerides (TG), total cholesterol (TC), low-density lipoprotein (LDL), and high-density lipoprotein (HDL) cholesterol were measured at baseline and at the end of the study.

**Results:**

Compared to the control group, the intervention group showed significant reductions in body weight and body mass index (BMI) (P = 0.017 and P = 0.026, respectively). There was a significant reduction in FBG (P < 0.001) and an increase in QUICKI (P = 0.022) in the intervention group in comparison with control, though QUICKI lost significance after adjusting for confounders. Both groups experienced significant reductions in TC and LDL during 12 weeks, while TG levels decreased significantly only in the intervention group (P = 0.001). Between-group comparisons revealed a significant difference in TG (P = 0.037), and a near-significant difference in LDL (P = 0.054). After adjusting for confounders, lipid profile components, except for LDL, improved in the intervention group.

**Conclusions:**

This study showed that a 12-week free sugar-restricted diet can lead to weight reduction and improvements in blood glucose and lipid profile parameters in women with PCOS.

**Trial registration:**

IRCT20230615058489N1; Iranian Registry of Clinical Trials (https://www.irct.ir); Date of registration: June 25, 2023.

**Supplementary Information:**

The online version contains supplementary material available at https://doi.org/10.1186/s12902-026-02451-3.

## Introduction

Polycystic ovary syndrome (PCOS) is the most common endocrine cause of infertility in women, characterized by increased ovarian androgen biosynthesis, anovulation, and consequently, infertility [1, 2]. Depending on the diagnostic criteria used, PCOS affects approximately 6% to 20% of women of reproductive age, with peak incidence occurring between the ages of 20 to 40 [3]. PCOS is diagnosed based on criteria established by the European Society of Human Reproduction and Embryology (ESHRE) and the American Society for Reproductive Medicine (ASRM). According to this consensus, PCOS is defined by the presence of at least two of the following three features: (1) oligo- or anovulation; (2) clinical and/or biochemical evidence of hyperandrogenism; and (3) polycystic ovarian morphology (PCOM). PCOM is characterized by the presence of ≥ 12 follicles measuring 2–9 mm in diameter in each ovary [4]. The diagnosis and management of PCOS impose an estimated annual cost of $4 billion on the U.S. healthcare system, not accounting for the treatment of long-term complications such as infertility, type 2 diabetes, and cardiovascular disease [5]. Additionally, PCOS negatively affects patients’ quality of life and is associated with significant psychological and social problems [6, 7]. The syndrome increases the risk of long-term complications including infertility, endometrial cancer, obesity, hyperinsulinemia, type 2 diabetes, metabolic syndrome, dyslipidemia, and atherogenic cardiovascular diseases [8, 9].

Numerous hypotheses have been proposed regarding the pathophysiology of PCOS, suggesting complex interactions between various genes and proteins, modulated by epigenetic and environmental factors. Nonetheless, the disease trajectory and optimal treatment strategies remain incompletely understood. Insulin resistance (IR) has recently been identified as a key metabolic disturbance associated with PCOS [10], leading to the use of insulin-sensitizing agents such as metformin for its management [11]. However, gastrointestinal side effects of metformin often result in poor compliance [12]. Dyslipidemia is another common metabolic abnormality observed in these patients, which may result from IR [13]. However, some evidence suggests that dyslipidemia may also occur independently of IR [14]. Moreover, dyslipidemia may exacerbate the PCOS condition, contributing to a vicious pathophysiological cycle [15].

Previous studies have demonstrated that diets high in simple sugars, particularly those rich in fructose, can promote lipogenesis and fat accumulation, even in the absence of weight gain [16–18]. The intake of simple sugars contributes to increased fat mass and IR [19], thereby elevating the risk of metabolic syndrome, a condition closely associated with PCOS. Additionally, simple sugar consumption has been shown to increase IR indirectly [20]. These findings suggest that reducing simple sugar intake, through mechanisms such as reduced lipogenesis and improved insulin sensitivity, may serve as a potential therapeutic strategy in PCOS management.

Given that sustained weight loss is often difficult to achieve and maintain, modifying dietary composition may offer a more practical therapeutic approach for these patients. A free sugar-restricted diet, which limits the intake of glucose, fructose, and sucrose found in sweetened foods, beverages, and fruit juices, typically provides less than 3% of total energy from free sugars. According to the World Health Organization, free sugars comprise all monosaccharides and disaccharides added to foods or beverages by the manufacturer, cook, or consumer, plus sugars naturally present in honey, syrups, and fruit juices; WHO recommends limiting free sugar intake to below 10% of total energy, with additional benefit below 5% [21]. In the present study, the intervention diet excluded table sugar, sugar-sweetened beverages, fruit juices, honey, confectionery, and pastries, while naturally occurring sugars in whole fruits and dairy were permitted. While this dietary pattern has been examined in a limited number of studies in patients with non-alcoholic fatty liver disease (NAFLD), showing improvements in lipid profile, glycemic indices, and inflammatory markers [22], its effects in PCOS remain unclear.

In the light of this knowledge gap, the present study was designed to investigate the effects of a free sugar-restricted diet in women with PCOS, focusing on its impact on anthropometric measurements, lipid profile, and glycemic control.

## Methods and materials

### Study design and participants

This randomized controlled clinical trial was conducted to assess the effects of a free sugar-restricted diet in women with PCOS. The study took place at *Valiasr* Hospital in Tehran, Iran. A total of 96 women aged 18 to 45 years, diagnosed with PCOS by a gynecologist based on the Rotterdam criteria, were initially assessed for eligibility through convenience sampling. After applying inclusion and exclusion criteria, 74 eligible participants were enrolled and randomly assigned to either the intervention group (*n* = 37), which received a free sugar-restricted diet, or the control group (*n* = 37), which received a regular diet. The intervention lasted for 12 weeks. The allocation sequence was generated using computer-generated random numbers. Given the nature of the dietary intervention, participants and dietitians could not be blinded to group assignment; however, personnel who performed anthropometric measurements and laboratory analyses were blinded to group allocation.

Inclusion criteria were as follows: (1) willingness to participate in the study, (2) body mass index (BMI) > 25 kg/m², (3) diagnosis of PCOS within the previous 6 months, (4) intake of more than 10% of total daily energy from free sugars, (5) no other chronic or acute gynecological conditions such as uterine or ovarian cancers, (6) absence of liver disease, renal failure, heart failure, infectious or inflammatory diseases, thyroid disorders, diabetes, any malignancy, or hyperprolactinemia, (7) not pregnant or breastfeeding, (8) no adherence to weight loss or special diets during the past 3 months, (9) No use of corticosteroids, omega-3 supplements, or spironolactone. These agents were excluded because each is independently known to affect insulin sensitivity or lipid metabolism (spironolactone through endocrine pathways, corticosteroids through glucose and lipid metabolism, and omega-3 supplements through triglyceride-lowering effects), and their use would therefore confound the outcomes of interest.

Exclusion criteria included unwillingness to continue participation, non-adherence to the prescribed diet for more than two consecutive days, initiation of a new dietary regimen outside the provided recommendations, pregnancy during the study period, changes in medication or physical activity during the intervention.

All participants provided written informed consent prior to enrollment. The study was conducted in accordance with the Declaration of Helsinki. The study protocol was approved by the Ethics Committee of the Islamic Azad University, Science and Research Branch (approval code: IR.IAU.SRB.REC.1402.041; dated June 10, 2023) and was registered on the Iranian Registry of Clinical Trials (https://www.irct.ir) under registration number IRCT20230615058489N1 (Date of registration: June 25, 2023). This study was reported in accordance with the Consolidated Standards of Reporting Trials (CONSORT) guidelines. A completed CONSORT checklist is provided as an additional file (Supplementary file).

### Dietary intervention

Participants randomized to the intervention group received counseling on the free sugar-restricted diet from a registered dietitian, based on a single standardized set of instructions applied uniformly to all participants rather than individualized meal plans. Instructions specified exclusion of table sugar, sugar-sweetened beverages, fruit juices, honey, confectionery, and pastries, while naturally occurring sugars in whole fruits and dairy were permitted, with the goal of keeping free sugar intake below 3% of total energy. Counseling was delivered verbally; no printed food-exchange lists or written meal plans were provided. Following the baseline counseling session, adherence was reinforced through monthly follow-up contact (approximately three contacts over the 12-week intervention), delivered through a combination of in-person visits and telephone calls, during which the dietitian reviewed progress, answered questions, and encouraged continued adherence to the restriction. Participants in the control group received general dietary advice consistent with usual clinical care and were not given any instruction to limit free sugar intake; they continued their regular diet for the duration of the study.

### Data collection

Initial demographic information and 3-day dietary recalls were collected through in-person interviews at the beginning of the study. Weight was measured using a *Seca* digital scale with an accuracy of 100 g, while height was measured using a stadiometer to the nearest 0.5 cm, with participants standing barefoot. Waist circumference was measured to the nearest 0.5 cm using a flexible tape at the narrowest point between the last rib and the iliac crest. Hip circumference was measured at the widest part of the hips. BMI was calculated as weight in kilograms divided by height in meters squared (kg/m²). Waist-to-hip ratio (WHR) was calculated by dividing waist circumference by hip circumference.

Fasting blood samples (7 mL) were obtained from the antecubital vein after 12–14 h of overnight fasting, both at baseline and at the end of the 12-week intervention. Blood was collected in EDTA-containing tubes and centrifuged at 2000 rpm for 10 min at room temperature to separate the serum. The serum samples were aliquoted into 1.5 mL microtubes and stored at − 80 °C until biochemical analyses were performed.

Serum triglycerides (TG) and high-density lipoprotein cholesterol (HDL-C) and Total cholesterol (TC) concentrations were measured using enzymatic photometric methods with commercial kits (MAN Company). Low-density lipoprotein cholesterol (LDL-C) was calculated using the Friedewald formula [23]. Fasting blood glucose (FBG) was measured via photometric methods using commercial kits (MAN Company, Iran). Serum insulin levels were determined by electrochemiluminescence (ECL) using Roche kits. IR was calculated using the homeostasis model assessment of insulin resistance (HOMA-IR) with the formula:

[insulin (µU/mL) × glucose (mg/dL)] / 405 [24]. Insulin sensitivity was calculated using the quantitative insulin sensitivity check index (QUICKI) with the formula: 1 / [log glucose (mg/dL) + log insulin (µU/mL)] [25]. To assess dietary intake including energy, carbohydrates, protein, total fat, fatty acids (SFA, MUFA, PUFA including omega-3 and omega-6), cholesterol, fiber, vitamin E, and vitamin C, three 24-hour dietary recalls (including one weekend day and two weekdays) were collected from each participant at baseline and at week 12 through a combination of face-to-face and telephone interviews. Between these two dietary assessment points, adherence was supported through the regular dietary counseling and follow-up contact described above and monitored qualitatively via the pre-specified exclusion criterion for non-adherence exceeding two consecutive days; a quantitative adherence rate (e.g., percentage of participants meeting the free-sugar target throughout the full 12 weeks, beyond the recall-based endpoints) was not separately calculated and is noted here as a limitation for future trials to address with more granular, continuous adherence tracking. The 24-hour dietary recall is a widely used, previously validated method for assessing dietary intake; no novel interview instrument was developed specifically for this study [26]. Dietary data were analyzed using Nutritionist IV (N4) software.

### Statistical analysis

All data were entered and analyzed using SPSS version 21 (SPSS Inc., Chicago, IL, USA). Quantitative variables were expressed as mean ± standard deviation (SD), and categorical variables as frequency (percentage). Between-group comparisons of categorical confounding variables like sex were assessed using the Chi-square test. The Kolmogorov–Smirnov test was applied to evaluate the normality of data distribution. For normally distributed variables, comparisons of means between the two groups were performed using the Independent Samples t-test, and within-group comparisons (pre- and post-intervention) were analyzed using the Paired Samples t-test. Additionally, to adjust for potential confounding variables with baseline and during intervention differences between groups, Analysis of Covariance (ANCOVA) was applied. A *P*-value < 0.05 was considered statistically significant.

Kolmogorov–Smirnov testing confirmed that all continuous outcome variables were normally distributed at both baseline and 12 weeks; means ± SD and parametric tests were therefore used throughout, as reported above.

A formal a priori sample size calculation was not performed before enrollment. To address this limitation, we conducted a retrospective power analysis using the study’s own completer data (independent-samples comparison, two-sided α = 0.05). For the primary glycemic outcome (FBG), the achieved sample (*n* = 35/33) provided > 99% power to detect the observed between-group difference (8.85 mg/dL, pooled SD 9.62), consistent with its high statistical significance (*P* < 0.001). For the primary anthropometric outcome (body weight), the achieved sample provided approximately 70% power to detect the observed difference (1.64 kg, pooled SD 2.71); a sample of approximately 43 participants per group would have been required for 80% power to detect this specific effect size. We note that this is a post-hoc calculation based on the observed effect size and is presented as contextual information rather than as a substitute for prospective power calculation; it indicates the study was well powered for the glycemic outcome but only moderately powered for the anthropometric outcome, and we have added this as an acknowledged limitation.

No correction for multiple comparisons was applied to the original analyses. To address this, we applied a Bonferroni correction (α = 0.05/3 = 0.0167) to the three primary, pre-specified confirmatory outcomes highlighted in the Abstract and Conclusion (body weight, FBG, and TG); all three ANCOVA-adjusted differences remained statistically significant (weight, *P* = 0.016; FBG, *P* < 0.001; TG, *P* = 0.012), although the margin for weight and TG was narrow. When the same correction is instead applied across all thirteen anthropometric, glycemic, and lipid variables tested by ANCOVA (α = 0.05/13 = 0.0038), only the FBG difference remains significant; the BMI, LDL, and HDL differences, in particular, would not survive this more conservative correction. We have added a corresponding note to the Discussion advising that findings beyond FBG (and, to a lesser extent, weight and TG) should be interpreted as hypothesis-generating and be confirmed in adequately powered future trials.

## Results

**Fig. 1:**
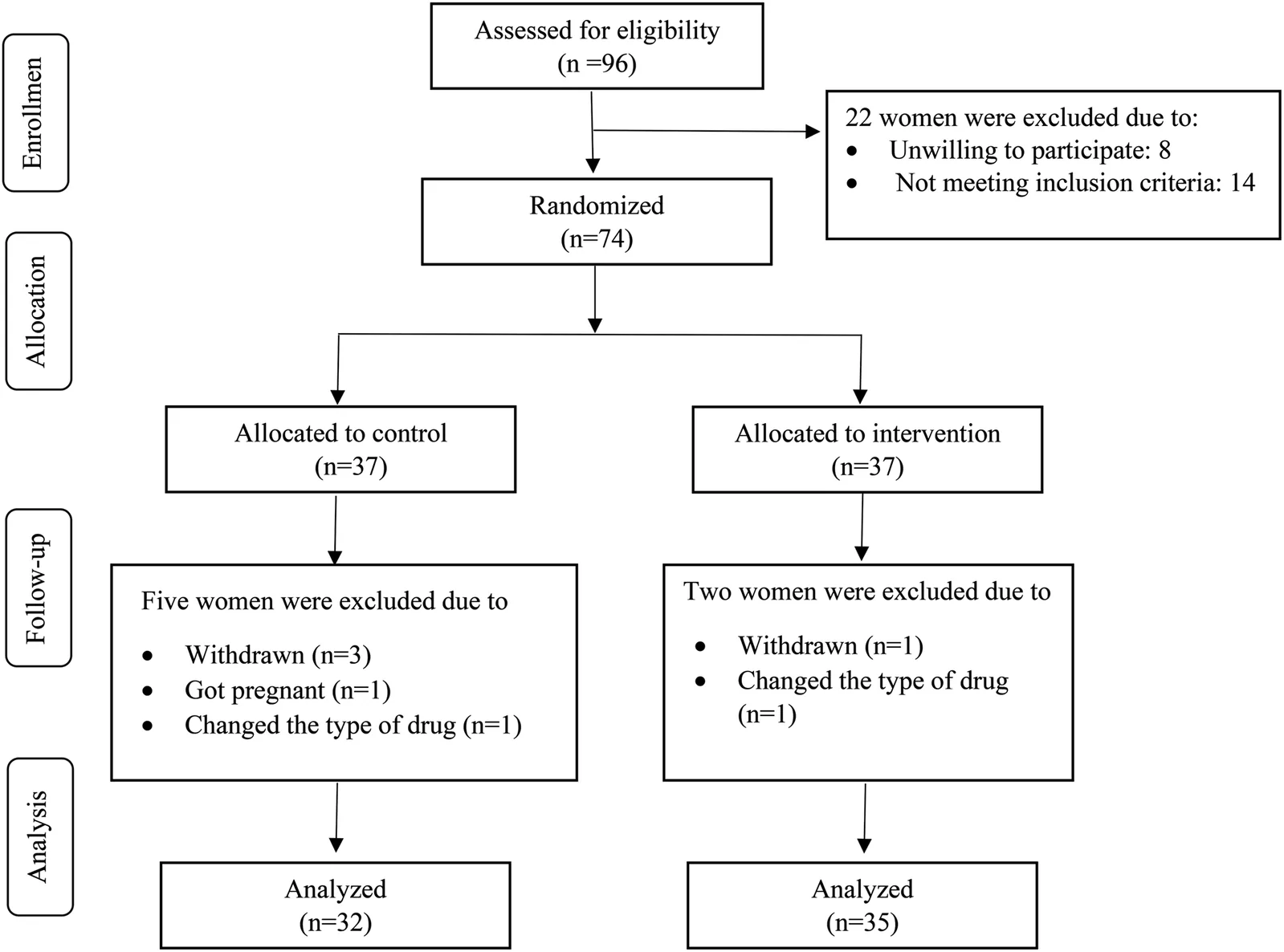
Flow diagram of patient’s recruitment

Out of 74 patients who met the inclusion criteria, 68 completed the study and entered the final analysis. The remaining six participants (three per group) were lost to follow-up, as detailed in Fig. 1; no imputation was performed for missing data, and analysis was conducted on a per-protocol basis restricted to participants who completed the 12-week intervention. A flow chart of study recruitment is provided in Fig. 1. The baseline characteristics of participants are presented in Table 1. The mean age of participants was approximately 27 years, with no statistically significant difference between the two groups. Additionally, marital status, smoking habits, duration of PCOS diagnosis, history of previous pregnancy, and use of medications such as metformin and anti-androgens did not differ significantly between groups. As shown in Table 2, dietary assessment indicated no significant differences in mean energy or macronutrient intake between the two groups at baseline or after the 12-week intervention. Medication use was not formally required to be stable for a minimum duration prior to enrollment; however, initiation of new medications or dose changes during the 12-week intervention was a pre-specified exclusion criterion, and metformin and anti-androgen use rates were statistically comparable between groups at baseline (Table 1), which limits, though does not eliminate, the potential for medication-related confounding.

**Table 1:** General characteristics of study participants by intervention and control groups

Characteristics	Intervention Group (*n* = 35)	Control Group (*n* = 33)	*p*-Value
Age (years)*	27.68 ± 5.51	27.75 ± 5.98	0.959
Marital status (%)			0.083
- Single	57.1%	57.6%	
- Married	42.9%	30.3%	
- Divorced	0%	12.1%	
Smoking (%)			0.966
- Yes	2.9%	3.0%	
- No	97.1%	97.0%	
History of previous pregnancy (%)			0.352
- Yes	5.7%	12.1%	
- No	94.3%	87.9%	
Duration of PCOS diagnosis (months)*	4.24 ± 2.31	3.45 ± 2.54	0.188
Metformin use (%)			0.432
- Yes	11.4%	18.2%	
- No	88.6%	81.8%	
Anti-androgen use (%)			0.670
- Yes	21.2%	17.1%	
- No	78.8%	82.9%	

*Quantitative variables are presented as mean ± standard deviation; qualitative variables are presented as percentages

Independent T-Test was used to compare quantitative variables, and Chi-Square test was used for qualitative variables

**Table 2:** Mean dietary intake in both groups at the beginning and end of the study

Nutrients	Intervention Group	Control Group	P**
**Energy (kcal/d)**			
Baseline	1734.6 ± 425.2	1633.17 ± 488.1	0.877
End of 12 weeks	1585.09 ± 376.5	1462.7 ± 351.04	0.118
Mean change	-115.4 ± 366.9	-127.88 ± 266.43	0.133
***P*** *	0.076	0.014	
**Carbohydrate (g/d)**			
Baseline	233.9 ± 96.8	190.8 ± 60.5	0.589
End of 12 weeks	197.2 ± 77.36	173.8 ± 65.5	0.201
Mean change	-29.5 ± 90.3	-18.46 ± 70.5	0.102
***P*** *	0.065	0.0.162	
**Protein (g/d)**			
Baseline	72.39 ± 34.91	60.02 ± 22.49	0.188
End of 12 weeks	63.28 ± 28.48	59.77 ± 30.3	0.634
Mean change	-9.71 ± 29.85	-0.31 ± 26.22	0.093
***P*** *	0.067	0.948	
**Fat (g/d)**			
Baseline	78.04 ± 17.38	73.27 ± 17.72	0.955
End of 12 weeks	73.08 ± 19.69	67.85 ± 15.56	0.247
Mean change	-5.39 ± 17.36	-5.61 ± 12.69	0.271
***P*** *	0.079	0.022	
**Cholesterol (mg/d)**			
Baseline	201.68 ± 107.92	202.80 ± 149.02	0.960
End of 12 weeks	196.14 ± 111.28	198.82 ± 136.61	0.931
Mean change	-2.74 ± 112.35	1.03 ± 159.72	0.972
***P*** *	0.823	0.972	
**Saturated fat (g/d)**			
Baseline	20.59 ± 14.60	17.28 ± 6.37	0.586
End of 12 weeks	17.71 ± 5.95	15.78 ± 4.99	0.170
Mean change	-3.11 ± 13.61	-1.65 ± 5.60	0.242
***P*** *	0.191	0.116	
**Monounsaturated fat (g/d)**			
Baseline	28.58 ± 6.85	27.64 ± 6.15	0.942
End of 12 weeks	26.99 ± 6.51	26.04 ± 5.98	0.551
Mean change	-1.72 ± 5.10	-1.62 ± 5.49	0.559
***P*** *	0.058	0.116	
**Polyunsaturated fat (g/d)**			
Baseline	19.97 ± 6.11	18.80 ± 5.52	0.486
End of 12 weeks	18.34 ± 6.71	17.96 ± 4.75	0.794
Mean change	-1.67 ± 5.01	-0.80 ± 4.80	0.417
***P*** *	0.061	0.365	
**Fiber (g/d)**			
Baseline	19.60 ± 6.83	19.54 ± 6.81	0.326
End of 12 weeks	18.58 ± 8.12	16.38 ± 7.50	0.265
Mean change	-1.11 ± 6.83	-2.91 ± 7.73	0.972
***P*** *	0.347	0.048	
**Vitamin C (mg/d)**			
Baseline	77.76 ± 22.71	66.73 ± 52.52	0.862
End of 12 weeks	80.18 ± 99.72	64.65 ± 58.72	0.459
Mean change	1.38 ± 90.53	-1.68 ± 34.34	0.482
***P*** *	0.927	0.655	
**Vitamin E (mg/d)**			
Baseline	26.98 ± 14.04	23.20 ± 7.56	0.175
End of 12 weeks	25.18 ± 13.04	23.54 ± 8.35	0.559
Mean change	-1.92 ± 6.50	0.32 ± 6.62	0.186
***P*** *	0.093	0.788	

* Variables are reported as mean ± standard deviation and were compared using the Independent T-Test

** *P-*values are reported respectively for baseline, end of study, and mean changes between groups

At baseline, no significant differences were observed between groups in anthropometric indices (Table 3). After 12 weeks, the intervention group showed significant reductions in weight, BMI, and waist circumference (*P* = 0.001, *P* = 0.001, and *P* = 0.014, respectively). Between-group comparisons also revealed significantly greater reductions in weight and BMI in the intervention group compared to the control group (*P* = 0.017 and *P* = 0.026, respectively). Although waist circumference decreased more in the intervention group, the difference between groups did not reach statistical significance (*P* = 0.073). These results remained unchanged after adjusting for potential confounders.

**Table 3:** Mean and standard deviation of anthropometric variables in study participants by intervention and control groups

Variables	Intervention Group	Control Group	P**	P***
**Height (cm)**	162.98 ± 4.58	162.31 ± 5.00	0.568	—
**Weight (kg)**				0.016
Baseline	65.37 ± 11.68	65.89 ± 10.42	0.847	
End of 12 weeks	63.54 ± 11.51	65.08 ± 9.69	0.561	
Mean change	-1.83 ± 2.91	-0.19 ± 2.48	0.017	
***P*** *	0.001	0.673		
**Body Mass Index (kg/m²)**				0.025
Baseline	24.58 ± 4.09	25.05 ± 4.07	0.639	
End of 12 weeks	23.91 ± 4.15	24.73 ± 3.69	0.403	
Mean change	-0.67 ± 1.009	-0.09 ± 0.96	0.026	
***P*** *	0.001	0.602		
**Waist Circumference (cm)**				0.081
Baseline	81.50 ± 11.22	82.16 ± 9.54	0.800	
End of 12 weeks	79.05 ± 10.32	81.56 ± 10.07	0.323	
Mean change	-2.03 ± 4.55	0.30 ± 5.57)	0.073	
***P*** *	0.014	0.770		
**Hip Circumference (cm)**				0.385
Baseline	101.75 ± 8.92	102.88 ± 8.08	0.593	
End of 12 weeks	100.23 ± 9.86	101.96 ± 7.42	0.425	
Mean change	-1.37 ± 4.27	-0.48 ± 2.60	0.325	
***P*** *	0.073	0.318		
**Waist-to-Hip Ratio**				0.218
Baseline	0.79 ± 0.06	0.79 ± 0.05	0.935	
End of 12 weeks	0.78 ± 0.06	0.79 ± 0.07	0.512	
Mean change	-0.007 ± 0.03	0.006 ± 0.04	0.192	
***P*** *	0.233	0.472		

Variables are presented as mean ± standard deviation

P*: Within-group comparisons using Paired T-Test

P**: Between-group comparisons using Independent T-Test

P***: Adjusted P-values based on ANCOVA considering baseline values and mean changes in energy intake

As shown in Table 4, there were no significant differences in glycemic markers between the two groups at baseline. After 12 weeks, the intervention group experienced a significant reduction in fasting blood glucose (FBG) (*P* < 0.001) and a significant increase in insulin sensitivity, as measured by QUICKI (*P* = 0.010). Between-group comparisons of changes over time revealed a significant decrease in FBG (*P* < 0.001) and an increase in QUICKI (*P* = 0.022) in the intervention group. After adjusting for baseline values and potential confounders, only the reduction in FBG remained statistically significant (*P* < 0.001). No significant within-group or between-group differences were observed for fasting insulin or HOMA-IR.

**Table 4:** Mean and standard deviation of glycemic and lipid parameters in study participants by intervention and control groups

Variables	Intervention Group	Control Group	P**	P***
**FBG (mg/dL)**				< 0.001
Baseline	96.31 ± 9.19	93.57 ± 6.50	0.163	
End of 12 weeks	87.51 ± 8.43	95.75 ± 10.52	< 0.001	
Mean change	-8.85 ± 9.85	1.84 ± 9.36	< 0.001	
***P*** *	< 0.001	0.274		
**Insulin (mg/dL)**				0.464
Baseline	13.97 ± 23.89	13.62 ± 25.24	0.953	
End of 12 weeks	9.83 ± 9.24	11.59 ± 12.55	0.513	
Mean change	-4.00 ± 19.68	-2.21 ± 21.27	0.722	
***P*** *	0.221	0.560		
**HOMA-IR**				0.228
Baseline	3.30 ± 5.60	3.17 ± 5.88	0.921	
End of 12 weeks	2.13 ± 1.94	2.80 ± 3.22	0.302	
Mean change	-1.17 ± 4.94	-0.41 ± 4.92	0.531	
***P*** *	0.167	0.634		
**QUICKI**				0.094
Baseline	0.49 ± 0.01	0.49 ± 0.10	0.245	
End of 12 weeks	0.51 ± 0.03	0.49 ± 0.20	0.087	
Mean change	0.01 ± 0.03	-0.002 ± 0.02	0.022	
***P*** *	0.010	0.667		
**TG (mg/dL)**				0.012
Baseline	98.88 ± 45.18	94.69 ± 51.59	0.722	
End of 12 weeks	85.88 ± 40.17	91.93 ± 45.43	0.565	
Mean change	-13.00 ± 19.98	-1.09 ± 25.58	0.037	
***P*** *	< 0.001	0.810		
**TC (mg/dL)**				0.346
Baseline	189.17 ± 40.07	185.57 ± 20.33	0.645	
End of 12 weeks	171.11 ± 28.25	172.75 ± 24.03	0.800	
Mean change	-18.05 ± 22.95	-11.90 ± 24.28	0.291	
***P*** *	< 0.001	0.009		
**LDL-C (mg/dL)**				0.015
Baseline	106.40 ± 27.43	106.36 ± 19.26	0.995	
End of 12 weeks	91.67 ± 13.78	98.50 ± 20.70	0.118	
Mean change	-15.35 ± 18.43	-7.00 ± 16.02	0.054	
***P*** *	< 0.001	0.019		
**HDL-C (mg/dL)**				0.024
Baseline	52.05 ± 6.71	51.87 ± 6.12	0.968	
End of 12 weeks	53.80 ± 5.80	49.15 ± 8.14	0.025	
Mean change	1.03 ± 8.40	-2.72 ± 8.22	0.070	
***P*** *	0.474	0.071		

Variables are presented as **mean ± standard deviation**

P*: Within-group comparisons via **Paired T-Test**.

P**: Between-group comparisons via **Independent T-Test**

P***: ANCOVA-adjusted P-values based on baseline values, BMI change, and energy intake

At baseline, there were no significant differences in serum lipid concentrations between groups. Both groups exhibited significant reductions in total cholesterol (TC) (intervention: *P* < 0.001; control: *P* = 0.009) and low-density lipoprotein (LDL) cholesterol (intervention: *P* < 0.001; control: *P* = 0.019) at the end of the study. No other significant changes in lipid profile components were observed. Triglyceride (TG) levels decreased significantly only in the intervention group (*P* = 0.001). Between-group comparisons showed a significant reduction in TG levels in the intervention group compared to the control group (*P* = 0.037), while the difference in LDL approached statistical significance (*P* = 0.054).

After adjusting for baseline confounders, significant changes in lipid profile components, including HDL (*P* = 0.012), LDL (*P* = 0.015), and TG (*P* = 0.024), were observed in the sugar-restricted group compared to the control group. No significant between-group difference was found for total cholesterol following adjustment.

## Discussion

This study is the first randomized controlled trial to investigate the effect of a free sugar-restricted diet on glycemic control, lipid profile, and anthropometric indices in women with PCOS. Both intervention and control groups showed significant reductions in total cholesterol and LDL levels. However, significant improvements in FBG, TG, weight, BMI, waist circumference, and insulin sensitivity were observed only in the group receiving the free sugar-restricted diet. Between-group comparisons also confirmed greater reductions in weight, BMI, FBG, LDL, TG, and an increase in HDL levels in the intervention group. Although insulin sensitivity improved significantly, this significance diminished after adjusting for confounders. The findings suggest that daily consumption of a diet low in free sugars can positively affect body weight, glycemic markers, insulin sensitivity, and lipid profile in this population.

The observed improvements in anthropometric parameters align with the findings of a recent Cochrane systematic review that included 15 clinical trials and 498 participants. This review reported that lifestyle interventions reduce weight and BMI, and subsequently improve reproductive outcomes such as the free androgen index (FAI), testosterone levels, sex hormone-binding globulin (SHBG), and hirsutism scores (Ferriman-Gallwey) in women with PCOS [27]. Other studies also suggest that modest weight loss (2–5% of initial weight) through lifestyle modification may enhance ovulation and menstrual regularity in PCOS patients [28–31]. Similar results were reported by Khodami et al. [22] in a 2022 RCT evaluating the effects of a free sugar-restricted diet in patients with NAFLD, where significant reductions in weight, BMI, waist, and hip circumference were found in the intervention group compared to those receiving a standard diet.

Beyond statistical significance, the magnitude of these changes appears clinically meaningful. The intervention group lost a mean of 1.83 kg (approximately 2.8% of baseline body weight) and 0.67 kg/m² in BMI over 12 weeks, placing it within the 2–5% weight-loss range that has been associated with improved ovulation and menstrual regularity in PCOS [27–30]. Similarly, the mean 8.85 mg/dL reduction in FBG (approximately 9% of baseline) moved the intervention group’s average glucose further from impaired fasting glucose thresholds. Although these effect sizes are modest in absolute terms, as expected for a non-pharmacologic and non-calorie-restricted dietary intervention, their direction and magnitude are broadly consistent with those reported for other lifestyle interventions that have demonstrated clinical benefits in women with PCOS. These findings therefore suggest that the observed effects may have practical as well as statistical significance.

Regarding lipid profile changes, our findings align with a 2010 study by Madero et al. [32], in which a fructose-restricted diet led to reductions in TG and total cholesterol but did not significantly affect HDL-C. This study involved 131 overweight and obese participants randomized to energy-restricted diets with either low or typical fructose intake. Jean A. Welsh et al. also found a positive association between added sugar intake and increased serum TG and TC levels [33]. Although significant changes in LDL-C and HDL-C were reported, these findings are inconsistent with the current study results. Differences in sugar types, intervention duration, and study populations may explain the discrepancies. The beneficial effects of sugar restriction on lipid metabolism may be mediated by reduced production of free fatty acids, leading to decreased hepatic VLDL and TG synthesis. Fructose promotes hepatic lipogenesis by increasing fructose-1-phosphate and stimulating fatty acid synthase (FAS) and acetyl-CoA carboxylase (ACC1), resulting in elevated acyl-CoA and VLDL production [17, 34]. Hence, limiting free sugar intake may counteract this pathway.

Beyond its effects on lipid and glucose metabolism, excess free sugar intake, particularly fructose, is also implicated in systemic inflammation and oxidative stress, both of which are recognized contributors to PCOS pathophysiology through worsening of IR and hyperandrogenism. Fructose metabolism generates reactive oxygen species and uric acid, which can activate pro-inflammatory pathways and reduce antioxidant capacity. Consistent with this mechanism, Khodami et al. [21] reported improvements in inflammatory markers alongside metabolic parameters after a free sugar-restricted diet in patients with NAFLD. Although the present study did not measure inflammatory or oxidative stress biomarkers, this represents a plausible additional mechanism linking free sugar restriction to the observed metabolic improvements and warrants direct investigation in future PCOS trials.

Madero et al. [32], who reported that a fructose-restricted diet improved glucose levels, insulin, and HOMA-IR in overweight and obese individuals, support glycemic outcomes in the present study. Similarly, Pollock et al. [35] showed a positive association between high fructose consumption and increased serum glucose and HOMA-IR among adolescents. Raben et al. [36] found that a sucrose-rich diet increased postprandial glucose, insulin, and lipids more than artificial sweeteners in overweight adults. These findings suggest that sugar type, dosage, and individual metabolic status influence outcomes. The likely mechanism underlying glycemic improvements involves reduced hepatic gluconeogenesis due to lower fructose availability, which decreases serum glucose levels and mitigates IR. Fructose-1-phosphate accumulation can activate IRS-1, exacerbating IR. Furthermore, increased MAPK8 (mitogen-activated protein kinase 8), a pro-inflammatory mediator, may contribute to the metabolic dysfunction and IR [37–40]. Therefore, sugar-restricted diets may improve hyperglycemia and insulin sensitivity by suppressing these pathways.

In our study, although FBG levels demonstrated a statistically significant improvement, no significant changes were observed in fasting insulin concentrations or HOMA-IR in either group. Several factors likely explain this dissociation. First, baseline insulin and HOMA-IR values showed very wide variability (SD exceeding the mean in both groups), which reduces statistical power to detect a between-group difference of the observed magnitude at this sample size. Second, the 12-week intervention period may have been too short for IR, which reflects more chronic and multifactorial dysregulation than fasting glucose, to respond measurably. Third, participants had relatively mild baseline insulin resistance (mean HOMA-IR approximately 3.2–3.3 in both groups), leaving less room for improvement than would be expected in a population with more severe IR. Improvement in FBG without a parallel change in HOMA-IR suggests that the diet may have acted predominantly through reduced hepatic glucose output rather than through a measurable change in peripheral insulin sensitivity over this timeframe. Larger, longer-duration trials enrolling women with more pronounced IR are needed to clarify whether free sugar restriction can meaningfully improve HOMA-IR in PCOS.

Since this is the first RCT to assess this intervention in women with PCOS, all participants underwent ultrasound assessments for ovarian morphology and were received regular dietary counseling and follow-up. Importantly, no pharmacologic interventions were implemented during the study. However, the inability to compare pre- and post-intervention ovarian status using ultrasonography, patient heterogeneity regarding disease severity, and restrictive inclusion criteria limited treatment generalizability. Additionally, since PCOS is primarily an endocrine disorder, this study did not include hormonal assessments (e.g., testosterone, SHBG, free androgen index, LH/FSH ratio), which limits our ability to draw conclusions about the intervention’s effect on the reproductive-endocrine features of PCOS rather than its metabolic manifestations alone. Finally, all participants were recruited from a single center in Tehran, Iran; because dietary habits, baseline free sugar intake, and genetic and environmental backgrounds vary across populations, these findings may not generalize directly to women with PCOS from other ethnic, geographic, or dietary contexts, and multi-center studies across diverse populations are needed to confirm external validity.

More broadly, our findings sit within a wider body of evidence on diet and cardiometabolic health in women. In a 25-year follow-up of the Women’s Health Study, greater adherence to a Mediterranean dietary pattern, another sugar-conscious, whole-food-based pattern, was associated with lower all-cause mortality in women, an association partly explained by improvements in cardiometabolic biomarkers similar to those assessed here [41]. Because women with PCOS carry an elevated lifetime cardiovascular risk [42], dietary strategies such as free sugar restriction may complement broader cardiovascular prevention efforts specifically tailored to women with metabolic disease, including type 2 diabetes. Beyond the biological effects of diet composition, psychosocial and behavioral factors also shape dietary behavior in women: stress and social determinants of health have been shown to influence eating behavior and cardiovascular risk in young women [43], and physical activity and sedentary behavior interact with diet to affect oxidative stress, inflammation, and metabolic outcomes in women across the lifespan [44]. These considerations suggest that free-sugar restriction is likely to be most effective as part of a broader, individualized lifestyle intervention that also addresses physical activity, stress, and social context in women with PCOS.

Overall, this 12-week intervention demonstrated that a free sugar-restricted diet improved serum levels of TG, LDL-C, HDL-C, and fasting glucose in women with PCOS. These results support the use of sugar-restricted diets alongside lifestyle modifications as a therapeutic approach in PCOS management. Given the absence of adverse effects, implementing a free sugar-restricted diet in clinical settings may enhance treatment outcomes in women with PCOS. Future studies with larger sample sizes, longer follow-up periods, and more comprehensive hormonal and inflammatory profiling are needed. Moreover, standardized imaging of ovarian morphology at baseline and post-intervention would help elucidate the diet’s effect on ovarian function.

## Conclusion

The findings of this randomized controlled trial indicate that adherence to a diet low in free sugars over a 12-week period significantly improves anthropometric parameters, glycemic indices, insulin sensitivity, and lipid profile in women with PCOS. These results underscore the potential of dietary modulation, specifically reduction in free sugar intake, as an effective, non-pharmacologic intervention for metabolic management in PCOS. The clinical implications of this study are substantial, as dietary interventions targeting free sugars are both feasible and sustainable, and may be implemented alongside standard lifestyle recommendations to enhance therapeutic outcomes. The absence of adverse effects further supports the safety and applicability of this approach.

However, given certain limitations, including heterogeneity in disease severity among participants and the inability to assess intra- and inter-group changes in ovarian morphology, further research with extended follow-up durations, larger sample sizes, and more comprehensive endocrine and imaging evaluations is necessary. Such studies will be critical to establishing the long-term efficacy and mechanistic underpinnings of low free sugar diets in the context of PCOS.

## Supplementary Information

Below is the link to the electronic supplementary material.

Supplementary Material 1

## Data Availability

Datasets used and/or analyzed during this study can be obtained from the corresponding author upon reasonable request.

## Abbreviations

ACC1: Acetyl-CoA Carboxylase 1
BMI: Body Mass Index
CRP: C - reactive protein
FAI: Free Androgen Index
FBG: Fasting Blood Glucose
FAS: Fatty Acid Synthase
HDL-C: High-Density Lipoprotein Cholesterol
HOMA-IR: Homeostatic Model Assessment of Insulin Resistance
IR: Insulin Resistance
IRS-1: Insulin Receptor Substrate-1
LDL-C: Low-Density Lipoprotein Cholesterol
MAPK8: Mitogen-Activated Protein Kinase 8
PCOS: Polycystic Ovary Syndrome
SHBG: Sex Hormone-Binding Globulin
TC: Total Cholesterol
TG: Triglycerides
VLDL: Very Low-Density Lipoprotein
